# Regulation of STIM1 and SOCE by the Ubiquitin-Proteasome System (UPS)

**DOI:** 10.1371/journal.pone.0013465

**Published:** 2010-10-18

**Authors:** Jeffrey M. Keil, Zhouxin Shen, Steven P. Briggs, Gentry N. Patrick

**Affiliations:** 1 Section of Neurobiology, Department of Biological Sciences, University of California San Diego, La Jolla, California, United States of America; 2 Section of Cell and Developmental Biology, Department of Biological Sciences, University of California San Diego, La Jolla, California, United States of America; University of California, Berkeley, United States of America

## Abstract

The ubiquitin proteasome system (UPS) mediates the majority of protein degradation in eukaryotic cells. The UPS has recently emerged as a key degradation pathway involved in synapse development and function. In order to better understand the function of the UPS at synapses we utilized a genetic and proteomic approach to isolate and identify novel candidate UPS substrates from biochemically purified synaptic membrane preparations. Using these methods, we have identified Stromal interacting molecule 1 (STIM1). STIM1 is as an endoplasmic reticulum (ER) calcium sensor that has been shown to regulate store-operated Ca^2+^ entry (SOCE). We have characterized STIM1 in neurons, finding STIM1 is expressed throughout development with stable, high expression in mature neurons. As in non-excitable cells, STIM1 is distributed in a membranous and punctate fashion in hippocampal neurons. In addition, a population of STIM1 was found to exist at synapses. Furthermore, using surface biotinylation and live-cell labeling methods, we detect a subpopulation of STIM1 on the surface of hippocampal neurons. The role of STIM1 as a regulator of SOCE has typically been examined in non-excitable cell types. Therefore, we examined the role of the UPS in STIM1 and SOCE function in HEK293 cells. While we find that STIM1 is ubiquitinated, its stability is not altered by proteasome inhibitors in cells under basal conditions or conditions that activate SOCE. However, we find that surface STIM1 levels and thapsigargin (TG)-induced SOCE are significantly increased in cells treated with proteasome inhibitors. Additionally, we find that the overexpression of POSH (Plenty of SH3′s), an E3 ubiquitin ligase recently shown to be involved in the regulation of Ca^2+^ homeostasis, leads to decreased STIM1 surface levels. Together, these results provide evidence for previously undescribed roles of the UPS in the regulation of STIM1 and SOCE function.

## Introduction

Stromal interacting molecule 1 (STIM1) is a type-I membrane, endoplasmic reticulum (ER)- resident protein and sensor of store-operated calcium entry (SOCE) [1]–[3]. In both excitable and non-excitable cells, SOCE is generally characterized by the process in which depletion of internal Ca^2+^ stores leads to an activation of plasma membrane Ca^2+^ channels, and subsequent refilling of internal stores [4], [5]. In addition to refilling stores, SOCE has been implicated in a myriad of diverse processes, including gene expression, apoptosis, and exocytosis (review by[6]). STIM1 is found distributed diffusely throughout the ER when Ca^2+^-stores are replete; but once stores are emptied, it redistributes into discrete, punctate clusters within the ER, at or near the plasma membrane [4], [7]. Whether STIM1 is inserted into the plasma membrane as a functional response to activated SOCE is still contested [4], [7]. STIM1 has also been shown to interact with the recently identified Ca^2+^-release activated Ca^2+^ channel (CRAC) component, OraiI, providing a link between store-depletion and plasma membrane CRAC channel activation[8]. In regards to the central nervous system, SOCE has been implicated in synaptic plasticity and neurite outgrowth [9], [10], but very little is known about STIM1 in neurons.

Protein modification via the covalent attachment of ubiquitin is one of the most commonly utilized regulatory processes in mammalian cells (review by [11]). Classically, ubiquitination is a process whereby target proteins can be marked for degradation by the proteasome. It is a multi-step enzymatic process, using three classes of enzymes (E1s, E2s, and E3s), and it involves the sequential transfer of ubiquitin from these enzymes to a lysine residue on the target protein. Specificity of the ubiquitination reaction depends on the later steps of the ubiquitination process. There are a significant, but limited, number of ubiquitin-conjugating enzymes (E2s), and a much larger number of ubiquitin ligases (E3s). Thus, the ubiquitination enzymes form a hierarchical cascade, where the substrate specificity of the overall ubiquitination reaction depends on the specific E2s and E3s that pair to ubiquitinate target substrates. Ubiquitination resulting in both degradative and non-degradative forms of protein regulation have been implicated in a myriad of cellular processes. Depending on the topology and length of the ubiquitin chain, changes in protein stability, interaction, and localization can be effected [12]. In the brain, the ubiquitin proteasome system (UPS) has long been implicated in a variety of neurodegenerative and neurological disorders. More recently, it has been shown to play a key role in normal neuronal function [13], [14]. Several studies have identified key synaptic proteins in mammals that are regulated in a UPS-dependent manner [15]–[20]. Given the emerging importance of the UPS in neurons, a logical step towards better understanding the scope of its function in neurons and at synapses would involve an examination of the neuronal targets of the UPS. Utilizing a genetic and proteomic approach we have isolated and identified novel ubiquitinated proteins and potential candidate UPS substrates from synaptically enriched rat brain fractions.

As STIM1 was identified as a novel candidate synaptic ubiquitinated protein in our proteomic screen, we sought to examine the role of the UPS in STIM1 and SOCE function. As very little is known about STIM1 in neurons, we first characterized the expression and subcellular distribution of STIM1 in rat brain tissues and dissociated hippocampal cultures by both biochemical and immunohistological methodologies. We report that STIM1 increases throughout development with stable, high expression in mature neurons. As in non-excitable cells, STIM1 is distributed in a membranous and punctate fashion in hippocampal neurons. A population of STIM1 is found at the surface of both mature and developing neurons, including growth cones. Thapsigargin (TG)-induced calcium store depletion promotes the rapid redistribution of STIM1 in hippocampal neurons. While STIM1 is expressed in the brain, extensive work has already shown that STIM1 regulates SOCE in non-excitable cells. Therefore, we examined the role of the UPS in STIM1 and SOCE function in HEK293 cells. While we find that STIM1 is ubiquitinated, its stability is not altered by proteasome inhibitors in cells under basal conditions or conditions that activate SOCE. However, we find that proteasome inhibitors significantly increase surface STIM1 levels and peak Ca^2+^ influx upon TG-induced SOCE. Furthermore, we find that the overexpression of POSH, an E3 ligase previously implicated in calcium homeostasis, decreases STIM1 surface populations. Together, these results provide compelling evidence for previously undescribed roles of the UPS in the regulation of STIM1 distribution and SOCE function.

## Results

### Characterization of STIM1 in neurons

**Figure 1:**
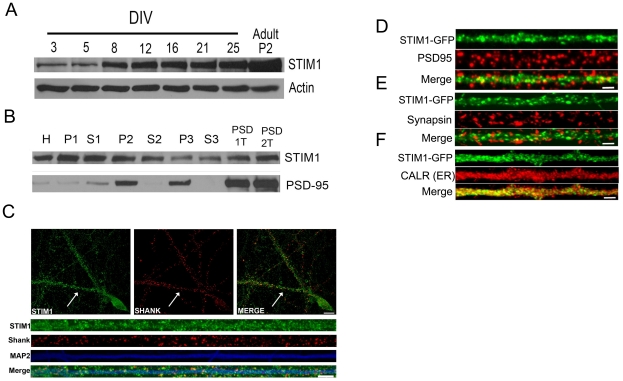
Characterization of STIM1 protein in neurons. (A) Lysates from cultured rat cortical neurons between days in vitro (DIV) 3 and DIV 25 were subjected to western blot analysis with α-STIM1 antibodies. Representative blots depicted from 3 independent experiments. (B) Western blot of adult rat brain fractions isolated by differential and density gradient fractionation probed with α-STIM1 antibodies (top panel) and α-PSD-95 (middle panel). Fractions are as follows: rat brain homogenate (*H*), crude synaptosome(*P2*), crude synaptosome supernatant (*S2*), lysed synaptosomal membrane fraction (*P3*), crude synaptic vesicle fraction (*S3*), single Triton X-100 extracted PSD (*PSD-1T*), and a double triton X-100 extracted PSD (*PSD-2T*). Representative blots depicted from 2 independent experiments. (C) Immunostaining of endogenous STIM1 protein in cultured rat hippocampal neurons (DIV 21). Neurons were immunolabeled with α-STIM1 (green), α-Shank (red), and α-MAP2 (blue) antibodies. Whole scale bars, 20 µm; dendrite scale bar, 10 µm. Cultured rat hippocampal neurons (DIV 21 and 4; A thru D and E thru F, respectively) were infected with Sindbis STIM1-GFP virion. Expression was allowed to continue for 12 hours prior to fixation, permeabilization, and immunolabeling with α- PSD-95 (D), α-synapsin (E), α-calreticulin (F) antibodies. Representative images depicted from >30 images acquired per co-stain. Scale bar  = 5 µm.

Although the physiological evidence for SOCE function in excitable cells has been shown [9], [21], very few reports have characterized STIM1 in neurons. STIM1 is expressed in various regions of the central nervous system including the cortex and hippocampus [22]–[24]. To better understand where STIM1 functions in neurons, we performed several biochemical and immunohistochemical experiments from neuronal tissues and dissociated neuronal cultures. We first determined the temporal protein expression profile of STIM1 from cultured, dissociated cortical neurons. As shown in Figure 1A, STIM1 protein levels are low in young developing cultures (DIV 3; days in vitro) and increases to relatively high stable levels in mature cultures (DIV 21 and 25). This expression pattern suggests STIM1 functions in both developing and mature neurons. To determine the subcellular distribution of STIM1, we fractionated rat brain by differential and density gradient fractionation methods as previously described [25], [26]. We found STIM1 to be present in both soluble and pellet fractions (Figure 1B). Moreover, STIM1 was present in fractions enriched for postsynaptic densities, suggesting that it may function at or near synapses (Figure 1B). We also characterized endogenous STIM1 in neurons by immunohistochemistry, finding STIM1 immunoreactivity distributed in a membranous and punctate fashion in both somatic and dendritic compartments of mature hippocampal neurons (Figure 1C). This is consistent with a recent report of STIM1 distribution in neurons [23]. Interestingly, we also found that a subpopulation of endogenous STIM1 punctate clusters co-localized with the postsynaptic density (PSD) protein marker Shank.

To further examine the distribution of STIM1 in hippocampal neurons, we expressed STIM1-GFP in neurons by Sindbis virus transduction. We created STIM1-GFP by fusing GFP in frame with STIM1 immediately after the signal peptide sequence (STIM1-GFP). When expressed in dissociated hippocampal neurons, STIM1-GFP is abundantly found in the ER (Figure 1F), and as with endogenous STIM1, is distributed in a membranous and punctate fashion (Figure 1D–F). In live-cell imaging experiments, we found that STIM1-GFP rapidly redistributes into larger punctate clusters in both somatic and dendritic compartments of hippocampal neurons treated with the Sarco/Endoplasmic Reticulum Ca^2+^ ATPase pump antagonist thapsigargin (TG) (Figure S1 and Movie S1). This is consistent with a recent study by Klejman and colleagues, where they found that YFP-STIM1 and ORAI1 redistribute into puncta-like structures in neurons treated with TG [23]. Furthermore, this indicates that the mechanism of STIM1 activation and TG-induced redistribution may be functionally relevant and similar to that found in non-excitable cells [4], [7]. In co-localization experiments, we also found STIM1-GFP co-localized with the postsynaptic and presynaptic protein markers, PSD-95 and synapsin, respectively (Figure 1D and E), which indicates a population of STIM1 is localized at or near synapses.

### STIM1 is present at the surface of hippocampal neurons

**Figure 2:**
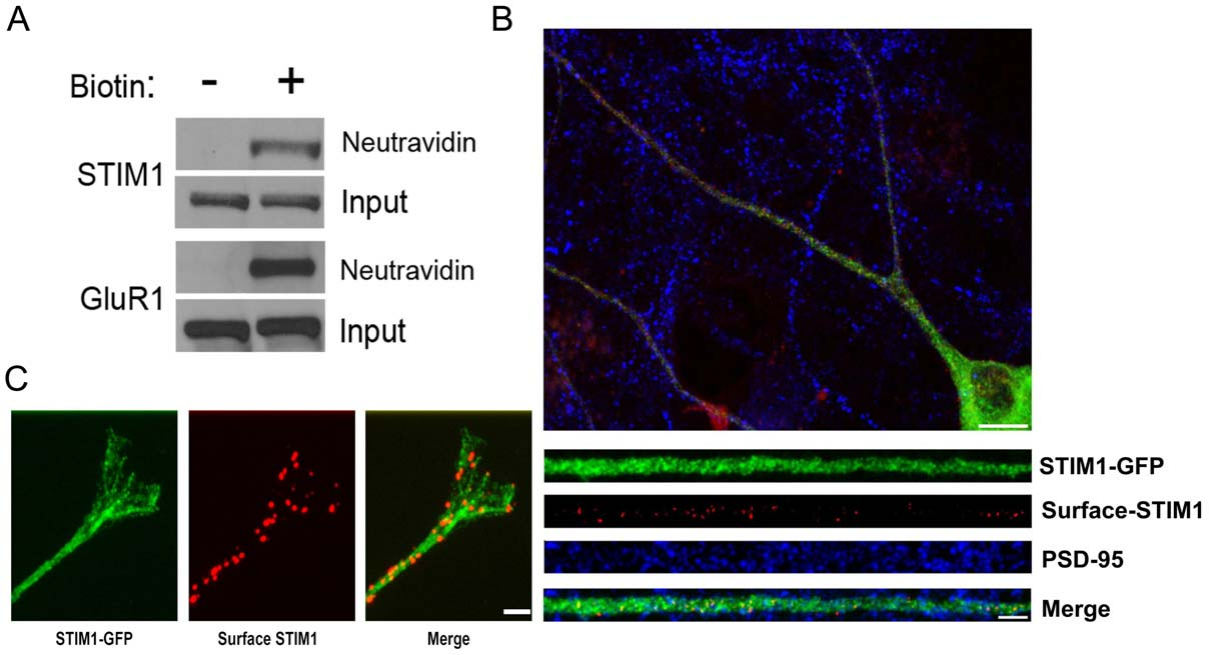
STIM1 is present at the surface of hippocampal neurons. (A) Cell surface STIM1 protein populations were examined biochemically by labeling cortical neurons (DIV >21) with NHS-LC-biotin followed by precipitation of the resulting lysates with neutravadin biotin binding beads. Precipitates were resolved by SDS-PAGE and analyzed via western blot with α-STIM1 (top) or α-GluR1 (bottom) antibodies. Representative blots depicted from 3 independent experiments. (B) Rat hippocampal neurons (DIV 21 and 4) were infected with Sindbis STIM1-GFP virion. Expression was allowed to continue for 12 hours. The cells were then live-labeled with α-GFP-Alexa 594 conjugated antibodies (red) prior to fixation, permeabilization, and immunolabeling with α-PSD-95 antibodies (blue). As depicted in (B–C), STIM1-GFP is present on the surface of dendrites and growth cones. Representative images depicted from >40 images acquired. Whole cell scale bar  = 20 µm; dendrite scale bar  = 10 µm; Growth cone scale bar  = 5 µm.

STIM1 is thought to physically interact with and activate SOCE channels by clustering in the ER directly beneath SOCE channels in the plasma membrane [1], [7], [27]–[29]. Far less understood, however, is a substantial population of STIM1that is able to bypass the ER retention machinery and traffic to the plasma membrane where it is thought to regulate Ca^2+^ entry through SOCs [3], [7], [30], [31]. In addition, it is speculated that STIM1 in the plasma membrane may mediate cell-cell interactions through its extracellular facing N-terminal domain [32], [33]. To examine whether STIM1 is also localized on the surface of neurons, we surface biotinylated mature dissociated high density cortical neurons and examined the neutravidin precipitates via western blot. We found STIM1 to be present in the biotinylated samples, while no immunoreactivity was present in non-biotinylated samples (Figure 2A). To verify the efficiency and specificity of our biotinylation procedure, we also probed for GluR1, a well-known cell-surface subunit of AMPA (alpha-**a**mino-3-hydroxy-5-**m**ethyl-4-isoxazole**p**ropionic **a**cid receptor) receptors. As with STIM1, GluR1 immunoreactivity was only present in the biotinylated samples (Figure 2A).

To confirm the plasma membrane insertion of STIM1 in neurons we expressed STIM1-GFP in cultured dissociated hippocampal neurons by Sindbis virus transduction, and after 12 hours of expression, the cells were live-labeled with anti-GFP antibodies. We observed a punctate surface fluorescent signal for STIM1-GFP in both somatic and dendritic compartments of mature hippocampal neurons (Figure 2B). Interestingly, many surface STIM1-GFP puncta were juxtaposed to PSD-95 clusters, indicating that STIM1-GFP may be integrated into the plasma membrane at or near synapses (Figure 2B). When expressed in immature cultures (DIV 4), we observed STIM1-GFP in growth cone filopodia extensions (Figure 2C), which is consistent with a recent study of endogenous STIM1 distribution in growth cones [34]. Interestingly, however, STIM1-GFP punctate clusters were also observed on the surface, many of which were at the tips growth cone extensions (Figure 2C). Together, these data suggest that STIM1 may function at the surface of neurons, at or near synapses in mature neurons, and in the growth cones of developing neurites.

### STIM1 is a candidate synaptic ubiquitinated protein

**Figure 3:**
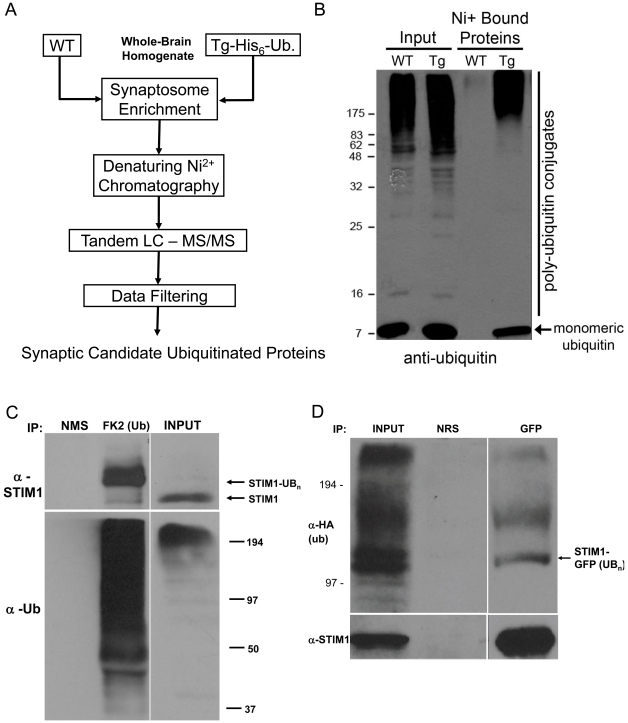
Isolation and identification of synaptic ubiquitinated proteins. (A) Schematized overview of proteomic screen for the isolation and identification of synaptic ubiquitinated proteins from mice expressing a hexahistidine-tagged ubiquitin GFP fusion transgene under the human UbC promoter (Tg-Ub mice). (B) Representative immunoblot depicting the ubiquitin immunoreactivity in Tg-Ub and WT (non-Tg) P2′ synaptosomal membrane total lysates or following nickel-affinity purification. (C) Adult rate brain P2′ synaptosomal membranes were solubilized and then immunoprecipitated with either α-STIM1 or α-ubiquitin (FK2). Immune complexes were resolved by SDS-PAGE and subjected to western blot analysis with α-STIM1 (top) or α-ubiquitin (bottom) antibodies Arrow indicates the relative mobility of unmodified STIM1. (D) HA-ubiquitin and STIM1-GFP were co-expressed in HEK293 cells. The resulting lysates were subjected to immunoprecipitation with either α-GFP or control serum (normal rabbit serum, NRS). Arrow indicates the relative mobility of monoubiquitinated-STIM1GFP. Representative blots depicted from 2 to 3 independent experiments for C and D.

We developed a genetic and proteomic approach to isolate and identify novel synaptic ubiquitinated proteins by mass spectrometry (Figure 3A). Crude, washed, synaptosomal membrane fractions (P2′) were isolated from whole brain tissues of mice expressing hexahistidine-tagged ubiquitin (Tg-His_6_-Ub mice). Cleavage of the fusion protein by endogenous enzymes produced moderate to high levels of the epitope-tagged ubiquitin that is detected both in monomeric form and conjugated to cellular proteins [35]. To isolate ubiquitinated proteins, while limiting contamination from non-ubiquitinated species, we performed the nickel affinity chromatography under denaturing conditions (6 M Gu-HCl). Similar fractions from non-transgenic (non-Tg) mice were used to control for non-specific binding of proteins to NiNTA affinity resin. Eluted proteins were then precipitated, solubilized, digested and then subjected to tandem mass spectrometry (LC-MS/MS) (See supplemental materials including Table S1 for complete details of the mass spectrometry methods). To verify the efficacy of our purification methods, we resolved a sample of the eluted proteins on SDS-PAGE and probed the blot with anti-ubiquitin antibodies. While similar amounts of ubiquitin immunoreactivity were found in the lysates from non-Tg and Tg-Ub samples, we only observed ubiquitin immunoreactivity (monomeric and conjugated forms) in Tg-Ub samples purified by nickel-affinity chromatography (Figure 3B). Using the spectral abundance of each identified protein from non-Tg and Tg-Ub samples we calculated the normalized Tg/non-Tg spectral ratio. Figure S2 is a graphical plot depicting the Tg/non-Tg spec ratio plotted against percent peptide coverage. We considered identified proteins with greater than 10% peptide coverage to be a synaptic candidate ubiquitinated protein (synCUP) if they also had a Tg/non-Tg spec ratio greater than 2. A total of 279 proteins met these criteria (Table S2 and S3). Several identified proteins with these criteria have been reported to be ubiquitinated in the literature. Indeed, the protein identified with the highest Tg/non-Tg spec ratio was ubiquitin, which further corroborated our isolation, identification, and group assignment strategies (Table S2).

STIM1 was identified as a candidate synaptic ubiquitinated protein by our proteomic strategies. To confirm this, we immunoprecipitated P2′ detergent-solubilized lysates with anti-ubiquitin conjugate specific antibodies (FK2) and probed the resulting blot with anti-STIM1 antibodies. STIM1 immunoreactivity was found in these precipitates (Figure 3C). As predicted for an ubiquitin modified protein, STIM1 showed decreased mobility in SDS-PAGE compare to unmodified STIM1 (Figure 3C). We also co-transfected HEK293 cells with STIM1-GFP and HA-tagged ubiquitin DNA constructs. The resulting lysates were immunoprecipitated with anti-STIM1 antibodies and resolved on SDS-PAGE. We found STIM1-GFP was covalently modified by the HA-tagged ubiquitin in HEK293 cells (Figure 3D). Taken together, these data indicate STIM1 is ubiquitinated.

### Proteasome inhibitors increase SOCE but STIM1 is not targeted for degradation by the proteasome

**Figure 4:**
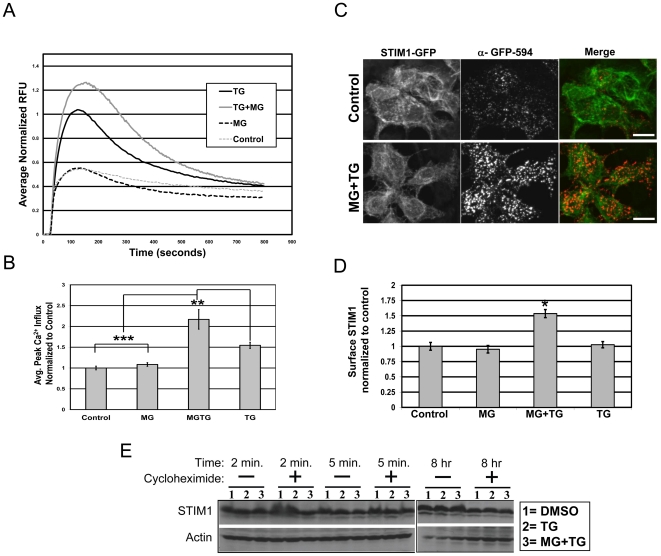
Effects of proteasome inhibition on store-operated calcium (SOC) influx, STIM1 subcellular localization and STIM1 stability. Potentiation of TG-induced calcium influx in HEK293 cells following pretreatment with the proteasome inhibitor MG-132 (10 µM). HEK293 cells plated in 96-well plates were loaded with the calcium indicator Fluo-4AM. Prior to loading the cells were pretreated with either vehicle (DMSO) or MG-132 (10 µM) for 1 h. The cells were then treated with 1 µM TG or vehicle (DMSO) for 30 min. Fluorescence was monitored by a FlexstationII 96-well fluorimeter, with Ca^2+^-re-addition (1.8 mM final) 5 minutes following baseline measurements. (A) Traces represent Ca^2+^-influx normalized to baseline, averaged over 12 wells per treatment. (B) Bar graph depicts averaged peak Ca^2+^ influx ± SEM. For statistical analysis, one-way ANOVA with *post hoc* Bonferroni's multiple-comparison test was used. *p<0.05; **p<0.01; ***p<0.001. (C) HEK293 cells expressing STIM1-GFP were treated as in (A), then live-labeled with α-GFP-Alexa 594 conjugated antibodies (red) prior to fixation. Scale bars, 10 µm. (D) Bar graph depicting the quantification of the STIM1-GFP surface levels from (D). Values were normalized to control. Mean values ± SEM are shown. * *p*<0.05, unpaired Student t-test. Represents the average of three independent experiments; 40 to 60 fields of cells per condition (15–25 cells per field). (E) HEK293 cells were treated in the absence (-) or presence (+) of cycloheximide (50 µg/mL) with either (1) DMSO, (2) 2 µM TG, or (3) 2 µM TG plus 10 µM MG-132 for the indicated times. Following treatments, protein levels were examined by western blot analysis with α-STIM1 or α-actin antibodies. Represents blots from 3 independent experiments.

Since STIM1 was identified in our screen for ubiquitinated proteins, we wondered if SOCE function was dependent on the UPS. Specifically, we examined TG-induced Ca^2+^ influx in cells treated with proteasome inhibitors. Since STIM1 was recently shown to be dispensable for SOCE in neurons [36], we examined if proteasome function was involved in SOCE in non-excitable cells. Specifically, HEK293 cells loaded with calcium indicator Fluo4-AM were imaged during TG-induced calcium influx in the presence of proteasome inhibitors. We found that cells pretreated with proteasome inhibitors had significantly increased TG-induced SOCE in HEK293 when compared to TG-treated alone cells (TG, 1.54±0.08; MG-132+TG, 2.16±0.23; MG-132, 1.08±0.04 fold change of control; *p*<0.001, one-way ANOVA) (Figure 4A and B). This data suggests that proteasome activity is required for normal TG-induced SOCE. Importantly, application of MG-132 alone had no effect on basal Ca^2+^ influx, indicating that store-depletion and activation of SOCs is required for the proteasome activity-dependent effect.

Previous studies have shown STIM1 functionality is dependent on both STIM1 levels as well as localization [1], [3]. Overexpression of STIM1 results in an increase in the amplitude of SOCE [1], whereas application of STIM1 antibodies specific to extracellular domains leads to an inhibition of CRAC channel current [3]. Thus, we next wanted to examine if this proteasome-dependent increase in SOCE correlated with a change in either STIM1 localization or stability. Under identical experimental parameters, we first examined the STIM1 surface population. HEK293 cells expressing STIM1-GFP were treated with proteasome inhibitor MG-132 (10 µM) prior to application of TG (1 µM) (Figure 4C). We found that STIM1-GFP surface levels were increased in cells pretreated with MG-132 (Figure 4D; TG + MG132, 1.54±0.07 fold change relative to control; n = 40 to 60 fields of cells per condition, 15-25 cells per field; *p*<0.05 compared to STIM1-GFP alone, unpaired Student t-test). This was in contrast to surface STIM1-GFP levels in cells treated with MG-132 or TG alone (MG-132, .95±0.06; TG, 1.02±0.05 fold change relative to control). Interestingly, using surface biotinylation, we also found that endogenous surface STIM1 levels increased in dissociated neurons in a similar manner as observed in HEK293 cells (Figure S3). While prior studies have shown that the surface expression of STIM1 is not a result of store-depletion [29], [37], this data indicates the UPS is a negative regulator of surface STIM1 expression, as TG-induced increased surface STIM1 levels were only observed in cells treated with proteasome inhibitors.

Since proteasome inhibition caused both an increase in SOCE and STIM1 surface levels, we next examined whether STIM1 was degraded by the proteasome. We compared the steady-state levels of endogenous STIM1 protein in HEK293 cells treated with TG (1 µM) in the presence of proteasome inhibitor, MG-132 (10 µM), and in the presence or absence of protein synthesis inhibitors (cycloheximide, 50 µg/mL). As seen in Figure 4E, we found no difference in STIM1 protein levels between vehicle-treated, TG-treated, or TG plus MG-132-treated cells in the presence or absence of protein synthesis inhibitors after 8 hours (Figure 4E). This suggests that STIM1 is not directly degraded by the proteasome under these conditions and that there likely exist other targets of the proteasome involved in SOCE function. Furthermore, it suggests that store-depletion and activation of SOCs does not cause a change in the turnover-rate of STIM1 in cells.

### POSH, a RING-finger ubiquitin ligase implicated in calcium homeostasis regulates STIM1 distribution in HEK293 cells

**Figure 5:**
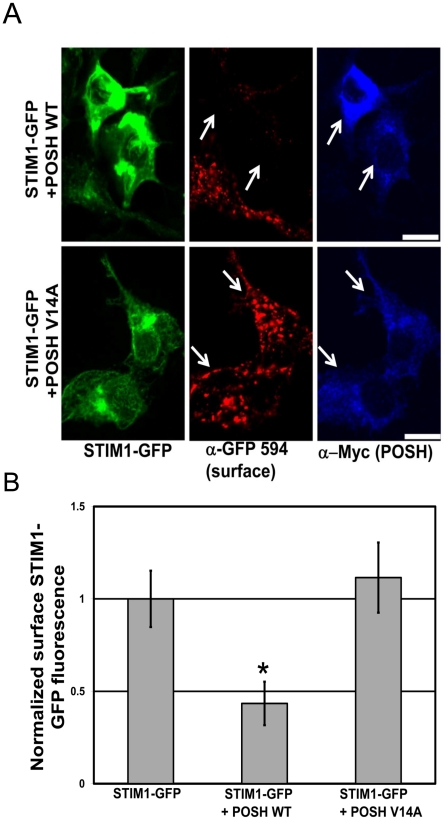
Overexpression of the E3 ligase POSH decreases surface STIM1-GFP levels. (A) HEK293 cells co-expressing STIM1-GFP (green), and WT or the RING-finger mutant (V14A) myc-tagged POSH constructs were live-labeled with α-GFP-Alexa 594 conjugated antibodies (red) prior to fixation, permeabilization and immunolabeling with α-Myc antibodies (blue). Co-expression of WT POSH dramatically reduced surface STIM1-GFP levels while the RING-finger mutant POSH^V14A^ had no effect on STIM1-GFP surface levels. Representative max z-projected confocal images are depicted. Scale bars, 10 µm. (B) Bar graph depicting the quantification of the STIM1-GFP surface levels in STIM1-GFP alone, or STIM1-GFP plus WT or catalytically inactive POSH expressing cells. Values were normalized to STIM1-GFP alone. Mean values ± SEM are shown. * *p*<0.05, unpaired Student t-test. Represents the average of three independent experiments; 40 to 60 fields of cells per condition (15–25 cells per field).

Since we did not observe any proteasome-dependent change in STIM1 stability, we wondered if ubiquitination might regulate its distribution in cells. Recently, the Really Interesting New Gene (RING)-finger containing ubiquitin ligase POSH (plenty of SH3′s) was found to be involved in calcium homeostasis [38]. POSH, which is associated with the trans-golgi network and was originally identified as a factor required for the targeting of HIV-1 to the plasma membrane [39], was shown to regulate calcium homeostasis by promoting the redistribution of Herp (homocysteine-inducible ER protein) to the ER [38]. Upon TG-induced ER calcium store depletion, POSH promotes Herp lys-63-linked polyubiquitination, which in turn promotes the redistribution of Herp to the ER [38]. Interestingly, POSH overexpression attenuates TG-induced calcium burst. Conversely, the inhibition of POSH by the expression of a RING-finger mutant, POSH^V14A^, or by RNAi, enhances this phenomenon [38]. Since POSH function is required for the maintenance of calcium homeostasis, presumably by controlling the distribution of regulatory molecules, we wondered if POSH might be involved in the regulation of STIM1 distribution. To examine this, we co-expressed WT myc-tagged POSH or POSH^V14A^ (a catalytically inactive variant) together with STIM1-GFP in HEK293 cells. We live-labeled HEK293 cells co-expressing myc-tagged POSH and STIM1-GFP with anti-GFP antibodies to detect surface STIM1-GFP and found that the overexpression of WT POSH dramatically reduced surface STIM1-GFP levels (STIM1-GFP alone, 1.0±0.16; STIM1-GFP plus POSH WT, 0.43±0.12; STIM1-GFP plus POSH V14A, 1.17±0.19; *p*<0.01 compared to STIM1-GFP alone, unpaired Student t-test, n = 40 to 60 fields of cells per condition,15–25 cells per field) (Figure 5A and B). This was dependent on POSH ligase activity, as the RING finger mutant, POSH^V14A^, had no effect on STIM1-GFP surface levels (Figure 5A and B). Interestingly, overexpression of POSH WT or POSH^V14A^ has no effect on the stability of STIM1 (Figure S4), which indicates its ability to decrease surface STIM1 levels does not involve the degradation of STIM1. Taken together, this suggests that POSH is a likely candidate E3 ligase involved in the regulation of STIM1 trafficking.

## Discussion

In this study, we characterized the ER calcium sensor and essential regulator of SOCE, STIM1, in neurons. STIM1 is expressed in immature neurons and its levels increase throughout development, remaining relatively high in mature neurons (Figure 1A). Upon biochemical fractionation of rat hippocampal tissues, we found STIM1 to be present in most subcellular fractions, including postsynaptic densities (Figure 1B). Furthermore, using immunohistochemistry we found that both endogenous STIM1 and ectopically expressed STIM1-GFP partially co-localize with synaptic proteins (Figure 1C–F). These expression and localization patterns suggest a role for STIM1 both in developing and mature neurons, at or near synapses. Using time-lapse confocal microscopy, we monitored STIM1-GFP redistribution in hippocampal neurons in response to TG-induced store depletion (Figure S1 and Movie S1). STIM1-GFP rapidly mobilizes into large punctate clusters in both somatic and dendritic compartments. This is similar to what has been observed for STIM1 redistribution in non-excitable cells [1], [29].

Calcium has been shown to play a critical role in growth cone dynamics, neurite outgrowth, and the development of synapses [40]. Interestingly, we also observed punctate STIM1-GFP clusters in dendritic growth cones when expressed in immature hippocampal neurons (Figure 2C). Moreover, a distinct population of STIM1-GFP was localized to the surface of dendrites, as well as at the tips of growth cone extensions (Figure 2B and C). Using surface biotinylation methods, we also detected endogenous STIM1 on the surface of mature neurons (Figure 2A). Together, these data suggest that there is a direct role for the physical insertion of STIM1 into the surface of neurons. It is intriguing to postulate a role for STIM1 in growth dynamics and neurite outgrowth, although we do not know if this population of STIM1 is functionally involved in SOCE. Furthermore, while there have been a few reports which have characterized STIM1 in neurons [22]–[24], our biochemical and immunohistochemical studies specifically highlight a role for STIM1 in mature neurons in dendrites at or near synapses and in developing neurons in growth cone compartments.

In this study, we have also uncovered a novel connection between the UPS and SOCE function. Foremost, in a proteomic screen for novel synaptic ubiquitinated proteins, we identified STIM1. We confirmed the ubiquitination of endogenous STIM1 in the brain (Figure 3C, D). Ubiquitinated STIM1 migrated on SDS-PAGE primarily as a single, higher molecular weight species, running approximately 8–10 kDa higher than STIM1. This would indicate STIM1 is modified by a single ubiquitin moiety. Interestingly, we found no evidence for STIM1 degradation by the proteasome (Figure 4E). We did, however, observe that proteasome function is involved in SOCE, as HEK293 cells pretreated with proteasome inhibitors had significantly increased TG-induced SOCE (Figure 4A and B). In contrast, application of proteasome inhibitors alone had no effect on basal Ca^2+^ influx which suggests that store-depletion and activation of SOCs are required for the proteasome activity-dependent effect. This indicates that one or more regulators of SOCE may be targeted for degradation by the proteasome under conditions of store-depletion and SOCE activation. The fact that surface STIM1-GFP levels are increased under similar conditions (Figure 4C and D) was surprising, and suggests that STIM1 surface levels are regulated by an unidentified target of the proteasome. Previous studies have already drawn a functional link between surface STIM1 and SOCE, wherein the blockade of surface STIM1 using N-terminal specific antibodies leads to a deficit in SOCE in HEK293 cells [3], [30]. Accordingly, our data would suggest that there are multiple levels at which the UPS regulates STIM1 and SOCE function. One scenario would involve the ubiquitination and proteasomal-independent regulation of STIM1. Indeed, we found that the co-expression of STIM1-GFP and POSH, an E3 ligase previously implicated in the regulation of regulatory molecules involved in calcium homeostasis [38], dramatically decreases surface STIM1-GFP levels while having no effect on the stability of STIM1 (Figure 5 and Figure S4). This was dependent on POSH ligase activity, as the RING finger mutant, POSH^V14A^, had no effect on STIM1-GFP surface levels. The second scenario involves the ubiquitin-dependent proteasomal degradation of other regulators involved SOCE function (Figure 4A and B) that likely converge to affect the distribution of STIM1 (Figure 4C and D).

In conclusion, we show that STIM1 distribution and SOCE function are dependent on UPS activity. While we find that STIM1 is ubiquitinated, STIM1 is not degraded by the proteasome. However, it is likely that intact proteasome function is required for a normal TG-induced SOCE response, as we have found that TG-induced SOCE is significantly increased in cells that were pre-treated with proteasome inhibitors. At one level this indicates that one or more proteins involved in SOCE are degraded by the proteasome in response to store-depletion. Therefore, both STIM1 ubiquitination and the degradation of other functionally related proteins are likely involved in the regulation of SOCE and will be important to study. We predict that there is a larger functional relationship between the UPS and SOCE that will be important to study further in both excitable and non-excitable cells.

## Materials and Methods

### Cell Culture

HEK293 cells were maintained and propagated in Dulbecco's modified Eagle's medium (DMEM) supplemented with 10% heat-inactivated fetal bovine serum and 2 mM glutamine in a humidified 95% air, 5% CO2 incubator at 37°C. Rat dissociated hippocampal neurons from postnatal day 1 were plated at a density of 45,000 cells/cm^2^ onto poly-D-lysine coated coverslips or glass bottom 35 mm dishes (Matek, Ashland, MA) and maintained in B27 supplemented Neurobasal media (Invitrogen, Carlsbad, CA) until 14 or 21 days or more as previously described [41]. High density rat cortical neurons from postnatal day 1 were plated onto poly-D-lysine 6 well dishes (∼500,000 cells per well) and maintained in B27 supplemented Neurobasal media (Invitrogen). All procedures were conducted in accordance with the National Institutes of Health Guide for the Care and Use of Experimental Animals and were approved by the University of California San Diego Institutional Animal Care and Use Committee (Approved Protocol ID# S04210).

### DNA and Sindbis constructs

STIM1-GFP was created by positioning EGFP (enhanced GFP; Clontech, Palo Alto, CA) at amino acid 23, immediately after the signal peptide sequence of mouse STIM1 (Accession: BC021644) purchased from Open Biosystems (Birmingham, AL). STIM1 signal peptide (amino acid 1–22) was PCR amplified and ligated into a unique NheI site in EGFP-C1. STIM1 (amino acid 23–685) was then amplified and mobilized via Bgl2-ApaI into the signal peptide-EGFP-C1. STIM1-GFP was further subcloned into the XbaI site of SinRep5 (Invitrogen). For production of recombinant Sindbis virions, RNA was transcribed using the SP6 mMessage mMachine (Ambion, Austin, TX), and electroporated into BHK cells using a BTX ECM 600 at 220 V, 129 Ω, and 1050 µF. Virion were collected after 24–32 hours and stored at −80°C until use. For Sindbis infection of hippocampal cultures STIM1-GFP virion were added directly to the culture media, with expression carried out for 12–18 hours prior to treatment and/or fixation. Wild-type myc-tagged POSH (Accession: AF030131; Tapon et al. 1998) was provided by Dr. Alan Hall through Addgene. POSH^V14A^ RING-finger mutant was mutagenized by a Quickchange (Stratagene, La Jolla, CA) protocol changing codon 14 from GTG to GCG. The following Quickchange primers were used: Forward, TGTCCGGCGTGTCTCGAG CGCCTTGATGCTTCTGCGA. Reverse, GAACAACCTAGAAAACCTC ACAGGCCGCACAGAGCTC.

### Antibodies, Reagents, and Drugs

FK2, α-ubiquitin conjugate (mAb, 1∶1000) antibody was purchased from Biomol (Plymouth Meeting, PA); α-ubiquitin (pAb, 1∶2000) antibody was purchased from Dakocytomation (Kyoto, Japan); α-synapsin antibody (pAb, 1∶1000) was purchased from Chemicon (Temecula, CA); α-PSD-95 antibody (mAb, 1∶1000) was purchased from Calbiochem (La Jolla, CA); α-GluR1 (pAb, 1∶1000 immunoblot) was purchased from Upstate Biotechnologies (Lake Placid, NY); α-STIM1 (mAb, 1∶1000 immunoblot; 1∶200 immunofluorescence) was purchased from BD Biosciences (Franklin Lakes, NJ); α-Calreticulin (pAb, 1∶1000) was purchased from StressGen (Victoria, BC, Canada); MAP2 (pAb, 1∶5000) was purchased from Abcam (Cambridge, UK). The following pharmacological reagents were used: MG132 from Peptide Institute (Osaka, Japan); Thapsigargin (TG) from Tocris-Cookson (Ellisville, MO).

### Western blot analysis and PSD-fractionation

For western blot analysis, all lysates were electrophoresed on SDS-polyacrylamide gels, transferred onto nitrocellulose membranes (Biorad, Hercules, CA), blocked with 5% fat-free milk in TBS/0.1% Tween20 (TBST), and incubated with primary antibodies in TBST +2%BSA overnight at 4°C. Membranes were then washed in TBST, and incubated with the appropriate HRP-conjugated secondary antibody in TBST +2%BSA, and developed with HyGlo ECL (Denville Scientific Inc, South Plainfield, NJ). PSD fractionation was performed as previously described [25], [26]. Briefly, adult female Sprague-Dawley rats (Harlan, Indianapolis, IN) were anesthetized with halothane (Baxter) and euthanized by rapid decapitation. Whole-brains were dounce homogenized (Teflon-glass) in 0.32 M Sucrose, 4 mM Hepes pH 7.4 plus Complete protease inhibitor cocktail (Roche, Indianapolis, IN). Synaptosomes were purified from crude membrane fraction (P2) by discontinuous sucrose density gradient centrifugation. PSD fractions were derived from synaptosomes by single (PSD-I) or double (PSD-II) extraction with 0.5% Triton X-100.

### Isolation of ubiquitinated proteins and Mass spectrometry

Crude washed membrane fractions (P2′) were prepared with whole-brains from transgenic 6X-His-ubiquitin-GFP mice kindly provided by Dr. Doug Gray of the University of Ottawa (Tsirigotis et al., 2001). P2′ fractions were then lysed in Buffer A (6 M guanidine-HCl, 0.1 M Na_2_HPO_4_/NaH_2_PO_4_, 10 mM imidazole, pH 8.0). Lysates were precipitated overnight via Ni-NTA resin (Novagen, Madison, WI). Following several washes in Buffer A, precipitates were eluted with 25 mM Tris-Cl pH 6.8, 25 mM EDTA. Eluates were precipitated with trichloroacetic acid (TCA) and pellets were washed in 100% acetone prior to processing form LC-MS/MS analyses. LC-MS/MS protocols are detailed in Methods S1.

### Immunoprecipitations

For all immunoprecipitations, cells were scraped and lysed in PB buffer plus 1% TritonX-100, 0.1% SDS, Complete protease inhibitors, 25 µM MG132, 100 µM NEM. Lysates were cleared at 18,000× g for 30′, and the resulting supernatant was incubated with 2 µg of antibody overnight. Immune complexes were precipitated using Protein A/G agarose beads (Pierce). Beads were washed 3 X in PB buffer and resuspended in 2X Laemelli sample buffer prior to western blotting.

### Surface biotinylation and live-labeling

For surface biotinylation, mature (>21 DIV) rat cortical cultures were washed once in PBS-MC (10 mM phosphate buffer, 2.7 mM KCl, 137 mM NaCl, 1 mM CaCl_2_, 0.5 mM MgCl_2_, pH 7.4) and incubated with 1 mg/ml NHS-LC-biotin (Pierce, Rockford, IL) in PBS-MC for 2 h at 4°C. Cells were then washed 2X in PBS-MC and lysed in precipitation (PB) buffer (10 mM NaPO_4_, pH 7.4, 5 mM EDTA, 5 mM EGTA, 100 mM NaCl) with 0.1% SDS, 1% Triton X-100, and complete protease inhibitors; rotated at 4°C for 30 min, and spun at 18,000× g. Cleared lysates were incubated with 30 µl of a 50% neutravidin bead slurry (Pierce, Rockford, IL) rotating for 2 h at 4°C. Beads were washed 4X in PB buffer and resuspended in 2X Laemelli sample buffer. Samples were resolved by SDS-PAGE and analyzed via western blot.

### Measurement of intracellular calcium

Fluo-4AM (Invitrogen, λex 495 nm; λem 516 nm) loaded HEK293 cells were prepared as previously described [2]. Briefly, HEK293 cells plated in 96-well plates were treated with 1 hour with 10 µM MG132 or vehicle (DMSO) and loaded with 5 µM fluo-4AM in culture medium for 30 min at 25°C. Cells were washed twice in calcium-free HBSS (115 mM NaCl, 1 mM KCl, 1 mM CaCl_2_, 1 mM MgCl_2_, 10 mM glucose, 10 mM HEPES pH 7.4) and placed in a FlexstationII^96^ fluorimeter (Molecular Devices). At T = 0s, the cells were treated with either 1 µM TG or vehicle (DMSO) for 5 min followed by re-addition of CaCl_2_ to a final concentration of 1.8 mM. Fluorescence at 515 nm was measured every 3 s after Ca^2+^ addition. Softmax Pro (Molecular Devices, Wokingham, UK) was used for data analysis. Traces of Relative Fluorescence Units (RFU) were normalized to control baseline and plotted from the point of calcium addition.

### Immunostaining and Confocal microscopy

For all imaging purposes, we used a Leica (Wetzlar, Germany) DMI6000 inverted microscope outfitted with a Yokogawa (Tokyo, Japan) Spinning disk confocal head, a Orca ER High Resolution B&W Cooled CCD camera (6.45 µm/pixel at 1X) (Hamamatsu, Sewickley, PA), Plan Apochromat 40X/1.25 na and 63X/1.4 na objective, and a Melles Griot (Carlsbad, CA) Argon/Krypton 100 mW air-cooled laser for 488/568/647 nm excitations. Confocal z-stacks were acquired in all experiments. For fixed cell imaging following treatments, hippocampal cultures (4–21 DIV) were fixed in 4% paraformaldahdye/4% sucrose (PFA) for 10 minutes at room temperature, and blocked and permeabilized in PBS-MC (phosphate buffered saline with 1 mM MgCl2 and 0.1 M CaCl2) with Triton X-100 (0.2%) and BSA (2%) for 15 minutes. Cells were incubated overnight at 4°C with primary antibody in PBS-MC and 2%BSA. Primary antibodies were visualized with appropriate secondary antibodies (1∶1000): Cy3 goat anti-chicken IgG (Jackson Immuno Research, West Grove, PA); goat anti-mouse Alexa 488, goat anti-rabbit Alexa 568, goat anti-mouse Alexa 568, and goat anti-mouse Alexa 647 (Invitrogen, Carlsbad, CA). Cells were then washed and coverslips were mounted in Aqua Poly/Mount (Polysciences, Warrington, PA). For live-labeling of surface STIM1-GFP, HEK293 cells were plated on poly-D-lysine (1 mg/ml) coated 35 mm glass coverslips and transfected with 1 µg of STIM1-GFP with Lipofectamine2000 (Invitrogen, Carlsbad, CA) according to the manufacturer's instructions. Prior to fixation and permeabilization, rabbit anti-GFP AlexaFluor 594 conjugated antibody (0.02 µg/µl final) was added directly to the cells in culture media and incubated 20 min at 37°C. Cells were washed 3X in PBS-MC, fixed in 4%PFA/4%Sucrose at room temp for 10 min, washed 3X in PBS-MC and mounted in Aqua/Poly mount.

### Image Analysis

All imaging was acquired in the dynamic range of 8 bit (0–255) with Simple PCI (Hamamatsu) imaging software. For quantitation of surface STIM1 immunofluorescence, max projected confocal z-stacks were analyzed with NIH ImageJ (Bethesda, MD). Surface STIM1 images were thresholded to a level 2X background and normalized to a total STIM1-GFP signal thresholded at 1.5X background. Surface fluorescence intensities were normalized to control groups and plotted as mean intensity ± SEM. Pooled, average values within each experiment were normalized to control and plotted as mean length ± SEM. Statistical significance was determined by unpaired Student's t-test or ANOVA with *post hoc* Bonferroni's multiple-comparison test as indicated.

## Supporting Information

Methods S1 Supplementary methods.
(0.03 MB DOC)

Table S1 Filtering criteria for autovalidation of database search results.
(0.03 MB DOC)

Table S2 Top synaptic ubiquitinated proteins by ratio.
(0.09 MB XLS)

Table S3 Unfiltered, list of proteins identified from Transgenic and non-Transgenic samples
(6.50 MB XLS)

Figure S1 Store-depletion induced rapid redistribution of STIM1-GFP in hippocampal neurons. Rat hippocampal neurons (DIV 18) were infected with Sindbis STIM1-GFP virion. Expression was allowed to continue for 12 hours prior to live-imaging in the presence of either vehicle or thapsigargin (TG; 2 µM final). Confocal images were taken at 2 min. intervals before (A) and after the addition of drugs (B). Representative max z-projected confocal whole cell images (A and B) and corresponding straightened dendrites (C) at indicated timepoints are shown. As shown, TG-induced ER calcium store-depletion rapidly promotes the redistribution of STIM1-GFP into large punctate clusters in both somatic and dendritic compartments. Whole-cell scale bars  = 20 µm; dendrite scale bar  = 10 µm. An accompanied full time-lapse video can be found in Movie S1.
(2.23 MB TIF)

Figure S2 Isolation of synaptic ubiquitinated proteins. Scatter plot (% peptide coverage vs. Tg/non-Tg spec ratio) of 385 distinct proteins identified via tandem LC-MS/MS. 279 proteins with greater than 10% peptide coverage and a Tg/non-Tg spec ratio greater than 2 was considered a synaptic candidate ubiquitinated protein (synCUP). Proteins with less than 10% peptide coverage plotted as black circles; proteins with a Tg/non-Tg spec ratio of less than 2 plotted as red circles; low abundant proteins with Tg/non-Tg spec ratio greater than 2 plotted as green circles; high abundant proteins with Tg/non-Tg spec ratio greater than 2 plotted as blue circles.
(0.81 MB TIF)

Figure S3 Proteaome inhibitors increase endogenous surface STIM1 levels in neurons in a TG-store depletion manner. (A) Dissociated cortical neurons were either treated with vehicle (DMSO) or MG-132 prior to the application of TG. The cultures were then labeled with NHS-LC-biotin followed by precipitation of the resulting lysates with neutravadin biotin binding beads. Precipitates were resolved by SDS-PAGE and analyzed via western blot with α-STIM1 (top) or α-GluR1 (bottom) antibodies. (B) Bar graph depicting the quantification of the STIM1 surface levels from (A). Values were normalized to control. Mean values ± SEM are shown. * p<0.05, unpaired Student t test. Represents the average of three independent experiments.
(0.40 MB TIF)

Figure S4 Overespression of POSH WTor POSH^V2A^ in HEK293 cells does not affect the stability of endogenous STIM1. HEK293 cells were transfected with either myc-tagged POSH WTor POSH^V2A^ for 24 hours. The resulting lysates were resolved on SDS-PAGE and analyzed via western blot with α-STIM1 (top) or α-myc (bottom) antibodies. Representative blot from 3 independent experiments.
(0.39 MB TIF)

Movie S1 Store-depletion induced rapid redistribution of STIM1-GFP in hippocampal neurons. Time-lapse video from Figure 3. Rat hippocampal neurons (DIV 18) were infected with Sindbis STIM1-GFP virion. Expression was allowed to continue for 12 hours prior to live-imaging of STIM1-GFP expressing neurons in vehicle and thapsigargin (TG; 2 µM final) containing imaging bath solutions to deplete ER calcium stores. Confocal images were taken at 2 min. intervals before and after the addition of 2 µM TG (TG added at T = 0). Scale bar  = 10 µm.
(4.15 MB AVI)
