# Supplementary material for: Regulation of STIM1 and SOCE by the Ubiquitin-Proteasome System (UPS)
Source: PLoS One. 2010 Oct 18;5(10):e13465. doi: 10.1371/journal.pone.0013465 (PMC2956693; doi:10.1371/journal.pone.0013465)
Supplement: Methods S1 — Supplementary methods. (0.03 MB DOC) [file pone.0013465.s001.doc]

**Supplemental Materials**

**Regulation of STIM1 and SOCE by the ubiquitin-proteasome system (UPS)**

**Jeffrey M. Keil^1^, Zhouxin Shen^2^, Steven P. Briggs^2^, & Gentry N. Patrick^1*^.**

**^1^ Section of Neurobiology, ^2^ Section of Cell and Developmental Biology, Department of Biological Sciences, University of California, San Diego, La Jolla, CA 92093-0347, USA**

**Supplemental Methods**

**Detailed mass spectrometry methodology**

Protein pellets were solubolized in 100uL of 1% RapiGest (Waters) and 50mM NH_4_HCO_3_ (pH8). The proteins were reduced and alkylated using 2 mM Tris(2-carboxyethyl)phosphine (Fisher, AC36383) at 95°C for 5 minutes and 5 mM iodoacetamide (Fisher, AC12227) at 37°C in dark for 30 minutes, respectively. The proteins were digested with 1 ug trypsin (Roche, 03 708 969 001) overnight. HCl was added to the mixture to precipitate RapiGest (pH1.4). Sample was incubated at 4°C overnight and then centrifuged at 16.1Kg for 15 minutes. Supernatant was collected and passed through a 0.22uM spin filter. The cleared solution was subject to Nano-LC-MS/MS analysis.

Automated 2D nanoflow LC-MS/MS analysis was performed using LTQ tandem mass spectrometer (Thermo Electron Corporation, San Jose, CA) employing automated data-dependent acquisition. An Agilent 1100 HPLC system (Agilent Technologies, Wilmington, DE) was used to deliver a flow rate of 300 nL min^–1^ to the mass spectrometer through a splitter. Chromatographic separation was accomplished using a 3 phase capillary column. Using an in-house constructed pressure cell, 5um Zorbax SB-C18 (Agilent) packing material was packed into a fused silica capillary tubing (200µm ID, 360 µm OD, 20 cm long) to form the first dimension RP column (RP1). A similar column (200µm ID, 5 cm long) packed with 5 µm PolySulfoethyl (PolyLC) packing material was used as the SCX column. A zero dead volume 1µm filter (Upchurch, M548) was attached to the exit of each column for column packing and connecting. A fused silica capillary (100µm ID, 360 µm OD, 20 cm long) packed with 5µm Zorbax SB-C18 (Agilent) packing material was used as the analytical column (RP2). One end of the fused silica tubing was pulled to a sharp tip with the ID smaller than 1µm using a laser puller (Sutter P-2000) as the electro-spray tip. The peptide mixtures were loaded onto the RP1 column using the same in-house pressure cell. Peptides were first eluted from RP1 column to SCX column using a 0 to 80% acetonitrile gradient for 150 minutes. Then the peptides were fractionated by the SCX column using a series of 8 salt gradients (20mM, 30mM, 40mM, 50mM, 60mM, 80mM, 100mM, 1M ammonium acetate for 20 minutes), followed by high resolution reverse phase separation using an acetronitrile gradient of 0 to 80% for 120 minutes.

Spectra were acquired on LTQ linear ion trap tandem mass spectrometers (Thermo Electron Corporation, San Jose, CA) employing automated, data-dependent acquisition. The mass spectrometer was operated in positive ion mode with a source temperature of 150 ^o^C. As a final fractionation step, gas phase separation in the ion trap was employed to separate the peptides into 3 mass classes prior to scanning; the full MS scan range was divided into 3 smaller scan ranges (300-800, 800-1100, and 1100-2000 Da) to improve dynamic range. Each MS scan was followed by 4 MS/MS scans of the most intense ions from the parent MS scan. A dynamic exclusion of 1 minute was used to improve the duty cycle.

The raw data was extracted and searched using Spectrum Mill (Agilnet, version A.03.02). MS/MS spectra with a sequence tag length of 1 or less were considered as poor spectra and discarded. The rest of the MS/MS spectra were searched against the IPI (International Protein Index) mouse protein database (version 3.14). The enzyme parameter was limited to full tryptic peptides with a maximum miscleavage of 1. All other search parameters were set to SpectrumMill’s default settings (carbamidomethylation of cysteines, +/- 2.5 Da for precursor ions, +/- 0.7 Da for fragment ions, and a minimum matched peak intensity of 50%). A concatenated forward-reverse database was constructed to calculate the in-situ false discovery rate (FDR). A total of 3,031 IPI proteins from the forward database were identified, while 102 proteins (3% protein FDR) from the reverse database were identified. Proteins with the same set or subsets of unique peptides were grouped into protein groups to minimize protein redundancy. There were 911 and 26 protein groups (3% FDR) identified from forward and reverse database, respectively.
