# Supplementary material for: Regulation of STIM1 and SOCE by the Ubiquitin-Proteasome System (UPS)
Source: PLoS One. 2010 Oct 18;5(10):e13465. doi: 10.1371/journal.pone.0013465 (PMC2956693; doi:10.1371/journal.pone.0013465)
Supplement: Table S1 — Filtering criteria for autovalidation of database search results. (0.03 MB DOC) [file pone.0013465.s002.doc]

Table S1. Filtering Criteria for autovalidation of database search results.

| mode | Protein score | 1+ peptide | 2+ peptide | 3+ peptide |
| --- | --- | --- | --- | --- |
| Protein Details | >20 | >9, >50% | >9, >50% | >11, >50% |
| Peptide | NA | >13, >50% | >13, >50% | >15, >50% |

ovie S1:

Supplemental Table 1)000000000000000000000000000000000000000000000000000000000000000000000000000000000000000000000000
