# Supplementary figures and images for: Regulation of STIM1 and SOCE by the Ubiquitin-Proteasome System (UPS)
Source: PLoS One. 2010 Oct 18;5(10):e13465. doi: 10.1371/journal.pone.0013465 (PMC2956693; doi:10.1371/journal.pone.0013465)

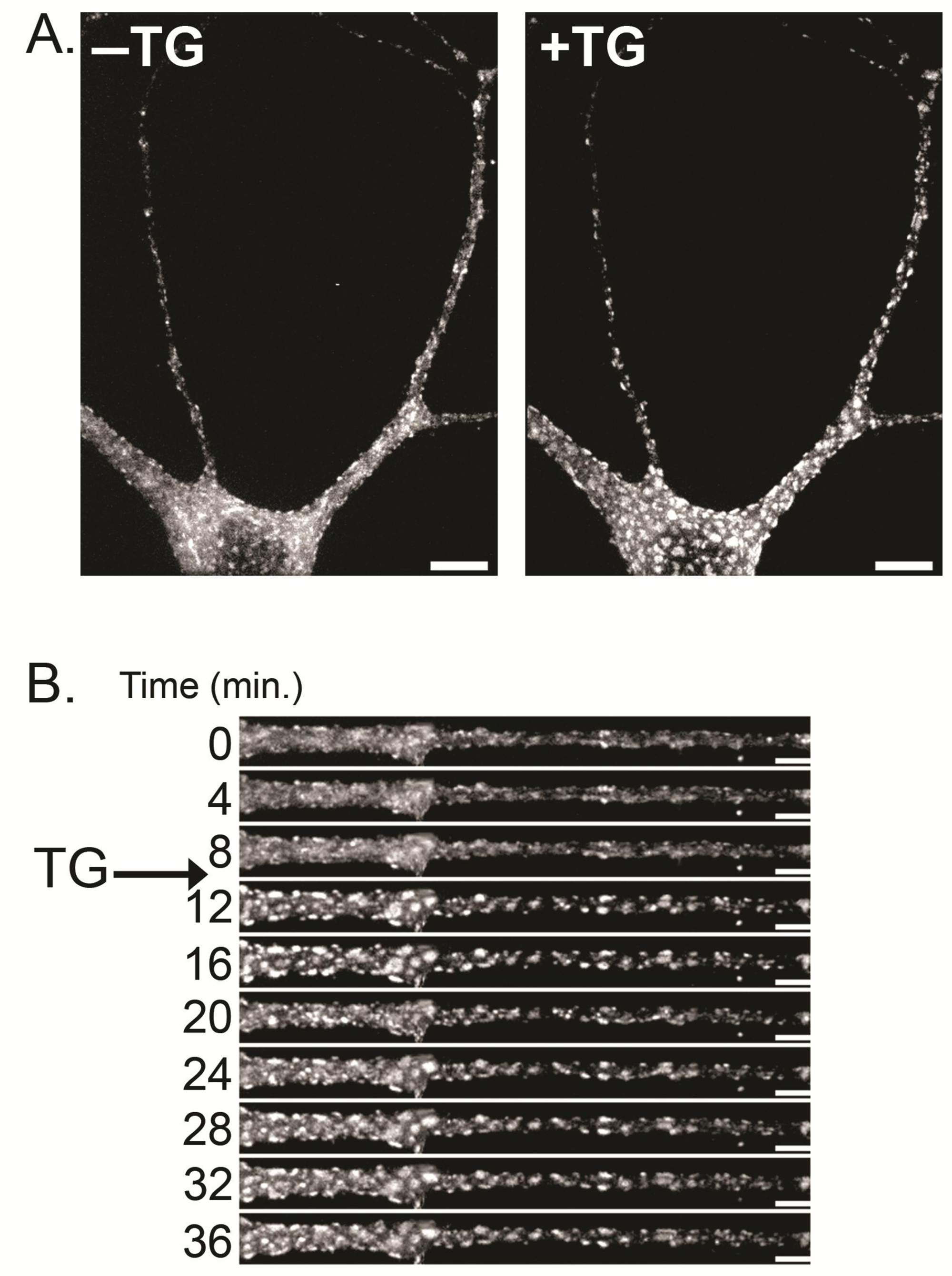

Supplement: Figure S1 — Store-depletion induced rapid redistribution of STIM1-GFP in hippocampal neurons. Rat hippocampal neurons (DIV 18) were infected with Sindbis STIM1-GFP virion. Expression was allowed to continue for 12 hours prior to live-imaging in the presence of either vehicle or thapsigargin (TG; 2 µM final). Confocal images were taken at 2 min. intervals before (A) and after the addition of drugs (B). Representative max z-projected confocal whole cell images (A and B) and corresponding straightened dendrites (C) at indicated timepoints are shown. As shown, TG-induced ER calcium store-depletion rapidly promotes the redistribution of STIM1-GFP into large punctate clusters in both somatic and dendritic compartments. Whole-cell scale bars = 20 µm; dendrite scale bar = 10 µm. An accompanied full time-lapse video can be found in Movie S1. (2.23 MB TIF) [file pone.0013465.s005.tif]

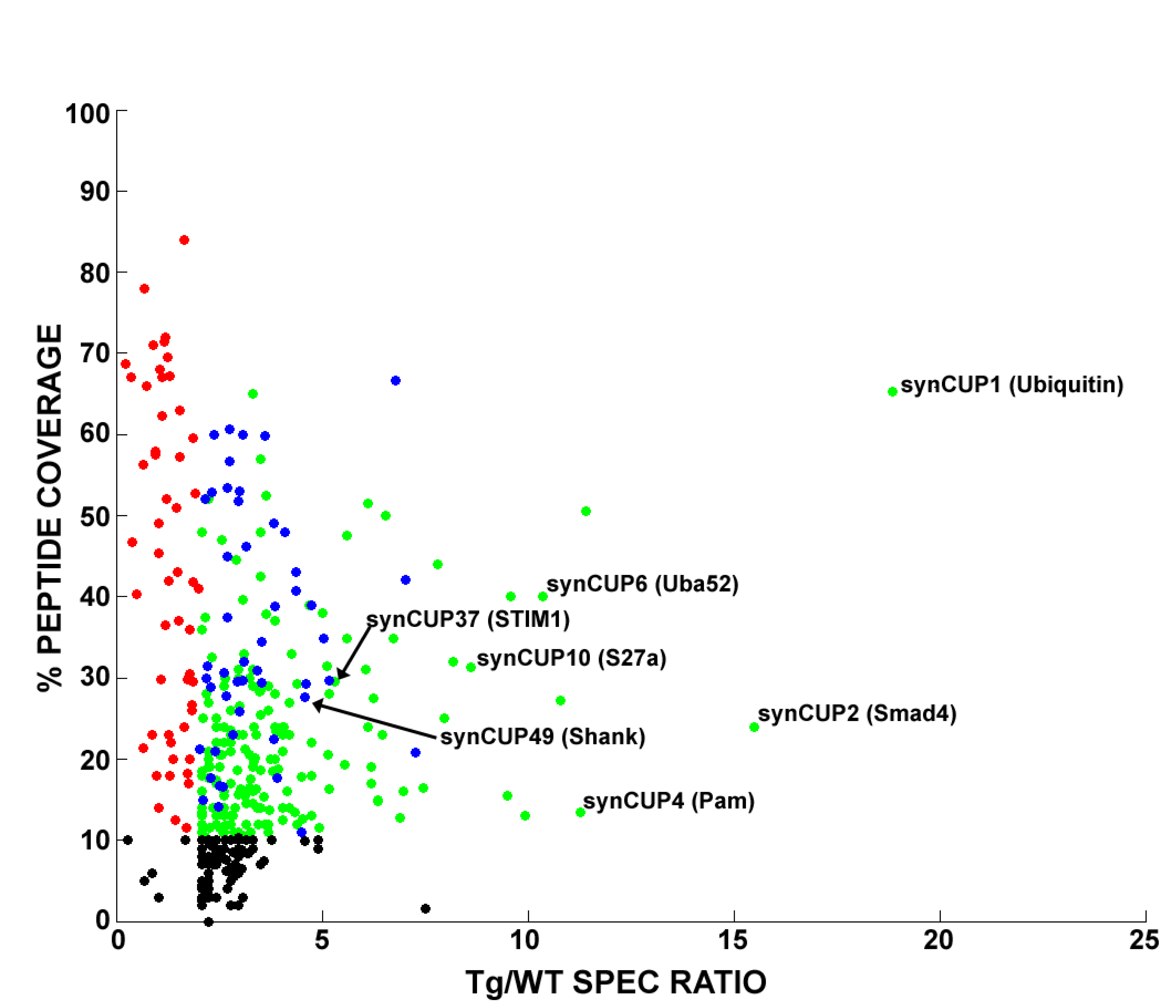

Supplement: Figure S2 — Isolation of synaptic ubiquitinated proteins. Scatter plot (% peptide coverage vs. Tg/non-Tg spec ratio) of 385 distinct proteins identified via tandem LC-MS/MS. 279 proteins with greater than 10% peptide coverage and a Tg/non-Tg spec ratio greater than 2 was considered a synaptic candidate ubiquitinated protein (synCUP). Proteins with less than 10% peptide coverage plotted as black circles; proteins with a Tg/non-Tg spec ratio of less than 2 plotted as red circles; low abundant proteins with Tg/non-Tg spec ratio greater than 2 plotted as green circles; high abundant proteins with Tg/non-Tg spec ratio greater than 2 plotted as blue circles. (0.81 MB TIF) [file pone.0013465.s006.tif]

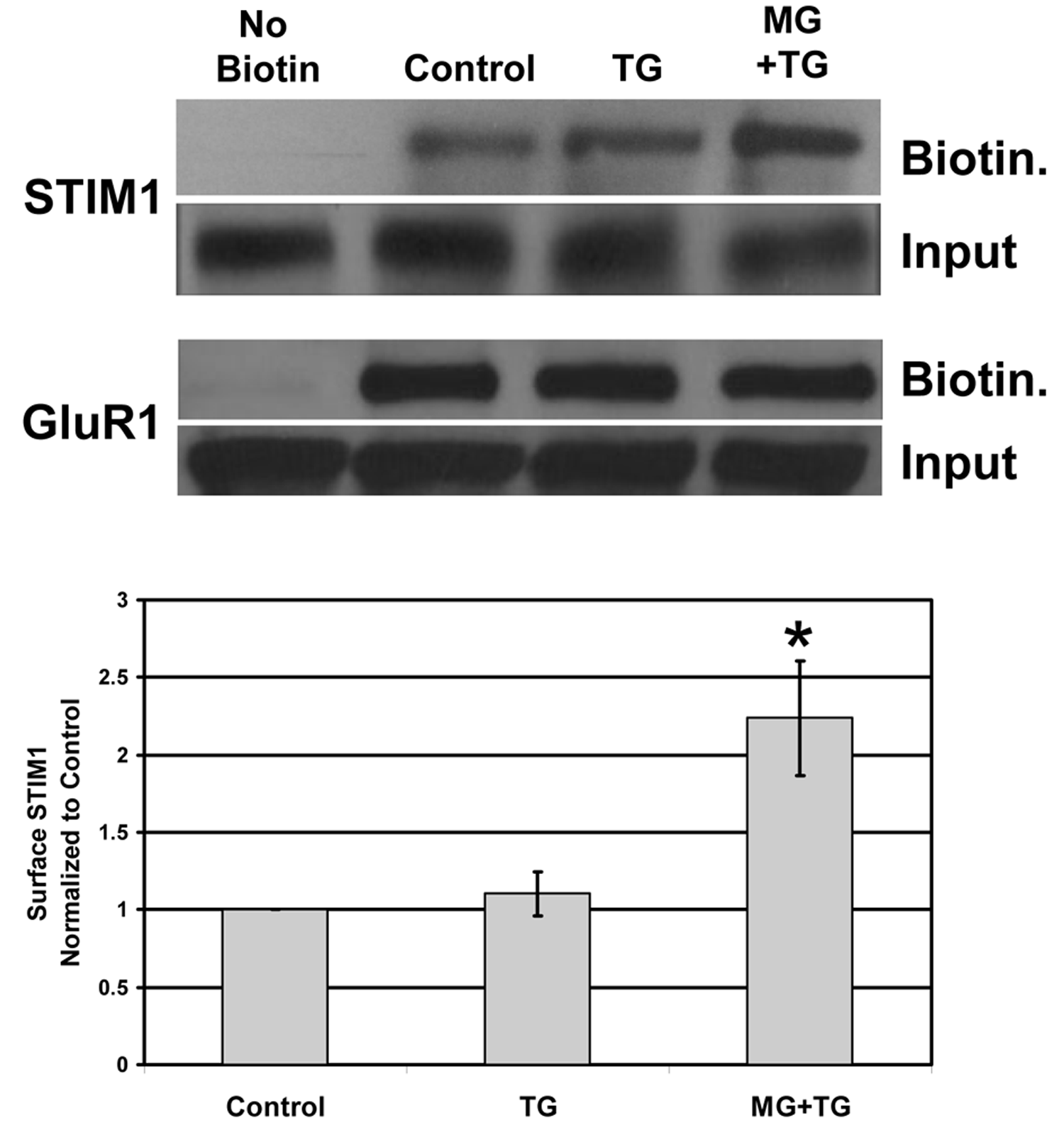

Supplement: Figure S3 — Proteaome inhibitors increase endogenous surface STIM1 levels in neurons in a TG-store depletion manner. (A) Dissociated cortical neurons were either treated with vehicle (DMSO) or MG-132 prior to the application of TG. The cultures were then labeled with NHS-LC-biotin followed by precipitation of the resulting lysates with neutravadin biotin binding beads. Precipitates were resolved by SDS-PAGE and analyzed via western blot with α-STIM1 (top) or α-GluR1 (bottom) antibodies. (B) Bar graph depicting the quantification of the STIM1 surface levels from (A). Values were normalized to control. Mean values ± SEM are shown. * p<0.05, unpaired Student t test. Represents the average of three independent experiments. (0.40 MB TIF) [file pone.0013465.s007.tif]

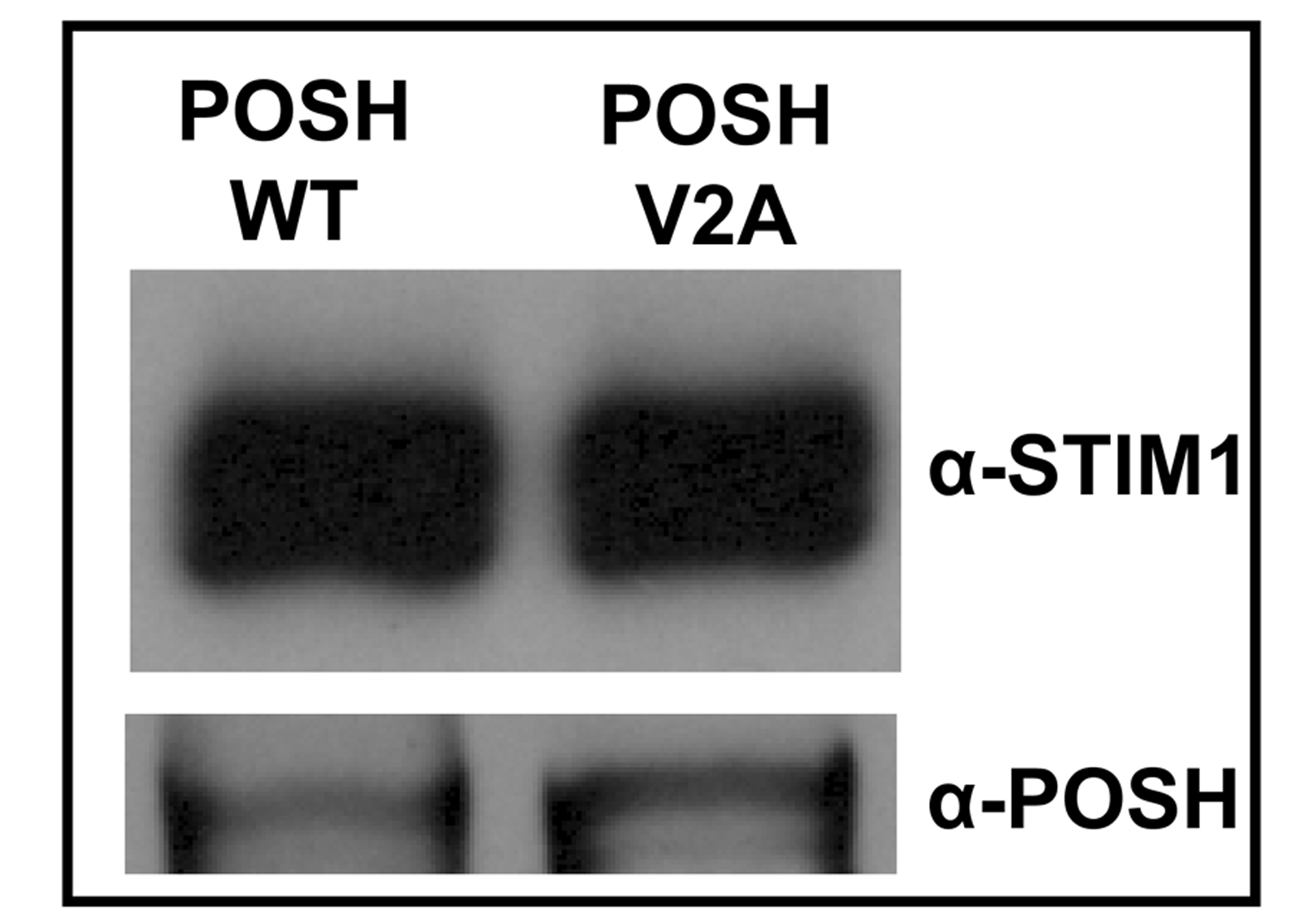

Supplement: Figure S4 — Overespression of POSH WTor POSHV2A in HEK293 cells does not affect the stability of endogenous STIM1. HEK293 cells were transfected with either myc-tagged POSH WTor POSHV2A for 24 hours. The resulting lysates were resolved on SDS-PAGE and analyzed via western blot with α-STIM1 (top) or α-myc (bottom) antibodies. Representative blot from 3 independent experiments. (0.39 MB TIF) [file pone.0013465.s008.tif]
